# EEG and fNIRS datasets based on Stroop task during two weeks of high-altitude exposure in new immigrants

**DOI:** 10.1038/s41597-024-03200-8

**Published:** 2024-04-08

**Authors:** Xiang Ji, Botao Bao, Lin Z. Li, Jiangbo Pu, Yu Lin, Xin Zhang, Zemeng Chen, Ting Li

**Affiliations:** 1 Institute of Biomedical Engineering, Chinese Academy of Medical Sciences and Peking Union Medical College, Tianjin, China; 2 School of optoelectronic science and engineering, University of Electronic Science & Technology of China, Chengdu, China; 3 Britton Chance Laboratory of Redox Imaging and Laboratory of Molecular Imaging, Department of Radiology, Perelman School of Medicine, University of Pennsylvania, Philadelphia, PA USA; 4 The Estee Lauder Companies, Melville, NY USA

**Keywords:** Optics and photonics, Psychology

## Abstract

Maintaining sufficient cerebral oxygen metabolism is crucial for human survival, especially in challenging conditions such as high-altitudes. Human cognitive neural activity is sensitive to fluctuations in oxygen levels. However, there is a lack of publicly available datasets on human behavioural responses and cerebral dynamics assessments during the execution of conflicting tasks in natural hypoxic environments. We recruited 80 healthy new immigrant volunteers (males, aged 20 ± 2 years) and employed the Stroop cognitive conflict paradigm. After a two-week exposure to both high and low-altitudes, the behavioural performance, prefrontal oxygen levels, and electroencephalography (EEG) signals were recorded. Comparative analyses were conducted on the behavioural reaction times and accuracy during Stroop tasks, and statistical analyses of participants’ prefrontal oxygen levels and EEG signals were performed. We anticipate that our open-access dataset will contribute to the development of monitoring devices and algorithms, designed specifically for measuring cerebral oxygen and EEG dynamics in populations exposed to extreme environments, particularly among individuals suffering from oxygen deficiency.

## Background & Summary

Oxygen metabolism is indispensable for human survival, and the brain, a pivotal organ supporting cognition, memory, and perception, is fundamentally reliant on a consistent oxygen supply^1–3^. It responds acutely to fluctuations in ambient oxygen levels^4^, presenting a significant challenge for individuals migrating from low-altitude regions to high-altitude locations^5–7^. Such migrations require adaptation to diminished brain oxygen levels resulting from increases in altitude^8–12^. This deficiency suppresses brain neural activity, creating an intriguing counterpoint in scenarios of controlled hypoxic/ischemic preconditioning^13,14^. The unique environment of high-altitude areas, characterized by low atmospheric pressure, induces a state of natural tissue hypoxia^15,16^. This leads to various physiological and psychological disruptions, such as shortness of breath, excessive fatigue, mental confusion, and headaches, underscoring the necessity for acclimatization preconditioning^17–22^. Motivated by this context, we embarked on a research endeavor focusing on a cohort of recently immigrated individuals exposed to high/low-altitude conditions for two weeks, serving as a model for high/low-altitude acclimatization. The data we present contribute to a deeper understanding of the interplay between altitude-induced physiological effects on brain function and the potential therapeutic applications of hypoxic preconditioning. In doing so, we aim to shed light on novel insights into neurology, cognitive science, and the optimization of human performance.

In recent years, there has been a gradual increase in publicly available datasets related to EEG and fNIRS, highlighting the growing significance of this dual-modality research approach. Anneke Hamann *et al*. induced mental fatigue through time on task during a 90-minute simulated flight, successfully obtaining and openly sharing data on EEG-fNIRS, behavioural performance, and self-report from 31 participants^23^. Similarly, Zeshan Shoaib *et al*. explored brain responses during fatigued driving under different coloured light stimuli^24^. Thien Nguyen *et al*. delved into brain functional connectivity by collecting EEG, EOG, and fNIRS data during rest and sleep states^25^. Furthermore, Michela Balconi’s team shared data on hemodynamics and brain event-related potentials during visual and auditory tasks^26^. Additionally, Wan-Chun Su *et al*. simultaneously recorded neural activity differences in motor execution, observation, and imagery conditions using EEG and fNIRS^27^. Prior to this study, our team contributed an open access dataset integrating EEG and fNIRS during the Stroop task^28^. These collective efforts underscore the comprehensive exploration of EEG and fNIRS applications in understanding diverse cognitive and neural processes.

In this paper, we conducted a statistical evaluation of the behavioural performance of 80 young immigrants at two distinct altitudes, namely Kashgar, China (71°20′ E, 35°37′ N, altitude: 4,500 m), and Aksu (79°43′ E, 39°28′ N, altitude: 800 m). To assess their cognitive responses, we employed the facial expression Stroop experiment^29,30^, consisting of 120 conflict stimuli. Our approach utilized a dual-modality instrument that combines functional near-infrared spectroscopy (fNIRS) and electroencephalography (EEG) to record neural activity. The key parameters subjected to analysis included reaction time (RT), accuracy (ACC), hemoglobin levels, EEG amplitude, power spectrum density, and time-frequency domain analysis. The findings from our study provide compelling evidence for individuals undergoing two weeks of high-altitude hypoxic preconditioning when confronted with conflicting stimuli. These observations contribute to the elucidation of the mechanisms underlying cerebral adaptation to hypoxia, offering robust support for such adaptations in the human brain. The implications of our study extend beyond the immediate context, shedding light on the broader understanding of how the human brain responds to high-altitude environments.

## Methods

### Instruments

**Fig. 1:**
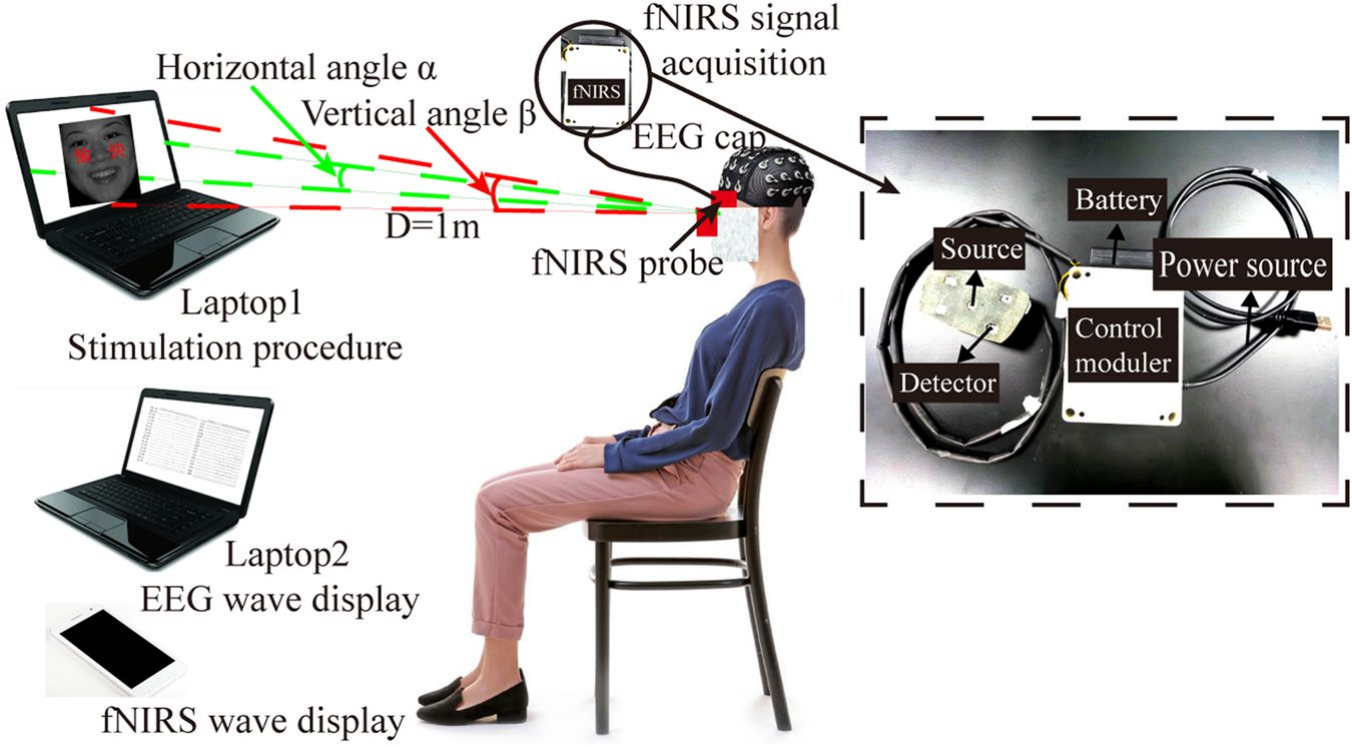
The experimental instruments. The dual-modality system of fNIRS-EEG was utilized for real-time monitoring of oxygen levels in the prefrontal lobe and cerebral neural activity.

We engineered a dual-modality instrument utilizing simultaneous fNIRS-EEG acquisition technology for the collection of neuro-EEG and prefrontal hemoglobin data (Fig. 1). Participants wore a 64-dry-electrode EEG cap (G.tec, g.Nautilus Research, that integrated with a fNIRS device while taking the test on laptop 1. The EEG device and fNIRS devices were programmed to concurrently acquire brain signals during the tests. The EEG data were recorded by laptop 2, and the fNIRS data were recorded on a mobile phone via Bluetooth. The details of the fNIRS device can be found in our previous research^31,32^. The EEG-fNIRS device enables us to monitor the activity area and oxygen consumption in a concurrent manner. Referring to the previously established paradigms of EEG experiments, the participants were comfortably seated at approximately 1 m from laptop 1 in a quiet environment throughout the experiment. The center of the laptop was aligned to the height of the participant’s eyes. The horizontal and vertical viewing angles were set at α = 3.2° and β = 3.1°, respectively.

### Procedures

**Fig. 2:**
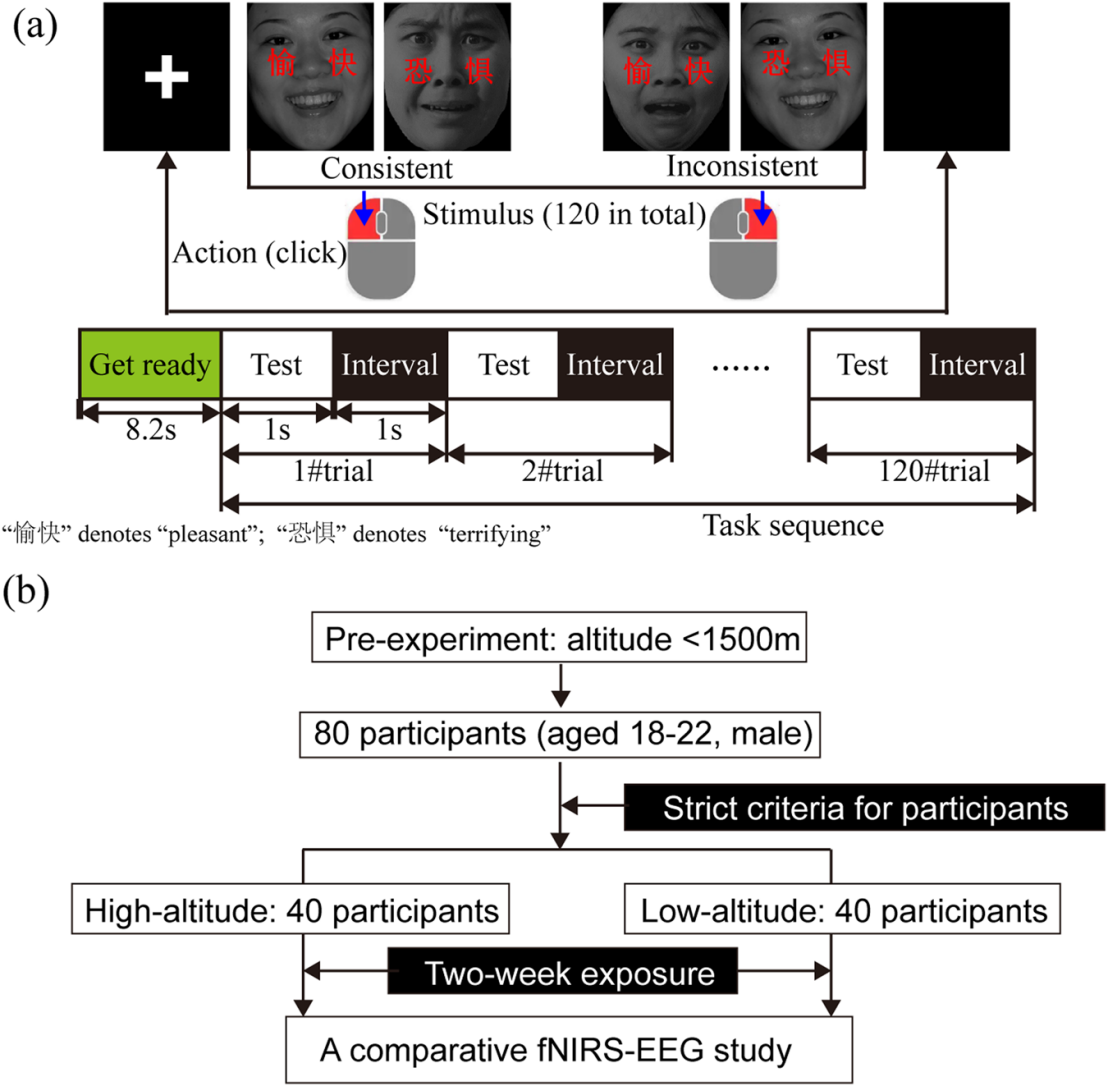
Experimental protocol and participant recruitment. (Image source: Chinese Facial Expression Picture System, Luo Yuejia).

The Stroop test with conflict stimulation comprised a preparation phase (8 s at the beginning) and an experiment phase (Fig. 2a). Brief experimental guidelines were also presented during this period. The formal experiment consisted of 120 task trials, each of which included a “Test” phase (1 s) and an “Interval” phase (1 s). The image gallery comprises two different types of images: those with pleasant or terrifying expressions, accompanied by Chinese characters for “pleasure” or “fear” (Image source: Chinese Facial Expression Picture System, Luo Yuejia). When the expression in the image aligned with the meaning of the Chinese characters, it was marked “Consistent” (abbreviated as “C”). Conversely, when there was a mismatch, it was marked as “Inconsistent” (abbreviated as “I”). If a “Consistent” stimulation was displayed, participants were instructed to quickly click the left mouse button. Conversely, when an “Inconsistent” stimulation was shown, they were to click the right mouse button. The stimulus sequence has been written in the dataset description file^33^ (figshare 10.6084/m9.figshare.c.6979581.v1).

### Participants

With the approval of the Ethics Review Committee of Tianjin University (TJUE-2021-019), we recruited 80 healthy participants (aged 18 to 22 years, male, Right-handed, Fig. 2b). Participants were sourced from college students engaged in social practice at the University of Electronic Science and Technology of China. Following thorough screening, it was ensured that their vision and hearing were normal, and they had no history of mental illness or other conditions that could potentially interfere with the experimental results. Prior to the experiment, all participants were informed and voluntarily signed the experimental consent form. We also took precautions during the informed consent process, assuring participants that their information would be treated confidentially and only used for research purposes. Ethical guidelines and institutional review board (IRB) approvals were followed diligently to ensure compliance with privacy standards. To eliminate any potential influence of the experimental sequence on decision-making accuracy, we employed a fully blinded approach to randomly divide the participants into two groups, each consisting of 40 individuals, who were then subjected to testing at high and low-altitude locations, respectively. During the two-week high/low-altitude acclimation period, participants underwent significant adaptive changes, maximizing benefits without causing undue stress or adverse health effects. Strict standards were upheld for environmental control during the EEG and fNIRS data collection process after the initial two weeks. This involved measures such as avoiding high-intensity magnetic fields in the same space, minimizing external stimuli like sound and light, and ensuring optimal human comfort.

Following relocation, the high-altitude group engaged in a 2-week preparatory practice aimed at facilitating sufficient physiological adjustments to altitude sickness. This practice facilitated increased red blood cell production, improved oxygen transport capacity, and enhanced oxygen utilization at the cellular level. These adjustments were essential to ensure the participants’ ability to perform the upcoming experimental tasks under normal conditions. To mitigate the divergence between the high-altitude and low-altitude groups, the low-altitude group was also mandated to complete the preparatory practice. Both groups commenced the experiments concurrently to ensure synchrony.

## Data Record

The open access dataset we have uploaded to Figshare^33^ comprises raw recordings of EEG, prefrontal cortex oxygenation (fNIRS data), and behavioural performance data, all saved in format with “.mat”. Additionally, there is a file named “Dataset Description.txt” that contains comprehensive information on all open access data, including data sampling rates, meanings of each parameter, methods for accessing the data, stimulus type sequences, and other pertinent details.

## Technical Validation

### Behavioural data processing

Reaction time (RT) and accuracy (ACC) are standard metrics utilized in the analysis of classical Stroop experiments. The processing and analysis of behavioral data are achieved using MATLAB R2022b and Origin 2021. By comparing the keypress records with the stimulus sequence, the response accuracy can be determined. The time difference between records of adjacent trials can be used to calculate the reaction time.

### fNIRS data processing

During the design phase of the fNIRS instrument, we addressed the issue of converting optical data into blood oxygen level data. The raw data provided on figshare has been transformed through the correction of the Beer-Lambert law, representing the change in hemoglobin levels in the prefrontal cortex. In order to eliminate inherent noise in the collected data due to factors such as blood flow dynamics, we need to apply band-pass filtering in the frequency range of 0.01 to 0.2 Hz. Based on the data received at 785 nm and 850 nm, we calculated the corresponding values for oxygenated hemoglobin and deoxygenated hemoglobin. Subsequently, these values were transmitted to a storage device for data preservation. The data exported from the storage device on figshare was segmented and extracted according to the time series of the experimental paradigm. Finally, participant data was averaged, and statistical analysis was conducted based on the stimulus types, categorized as “consistent (C)” or “inconsistent (I).”

### EEG data processing

**Fig. 3:**
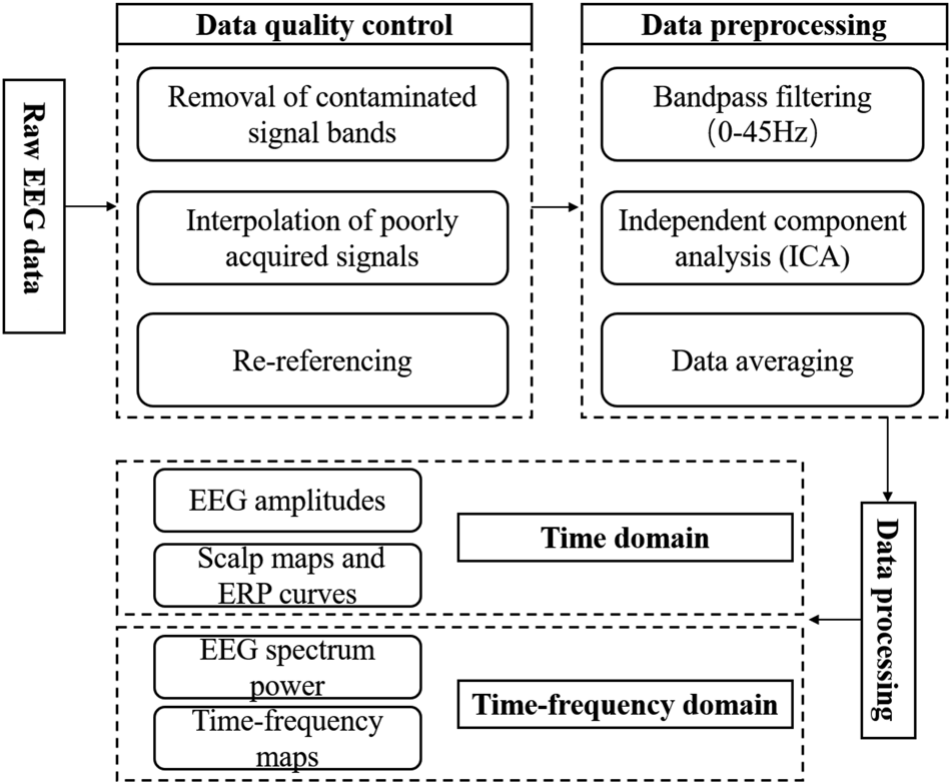
Schematic diagram of the processing process of EEG raw data.

The postprocessing of the EEG signals involved several steps, as Fig. 3 shows: 1. Removal of contaminated signal bands: We eliminated any contaminated signal bands present in each subject’s data. 2. Interpolation of poorly acquired signals: Electrodes with poorly acquired signals were interpolated to ensure complete coverage. 3. Re-referencing: All electrodes were re-referenced to enhance the quality of the EEG signals. 4. Bandpass filtering: A bandpass filter with a range of 0.1–45 Hz was applied to filter the EEG signals. 5. Artifact removal using independent component analysis (ICA): ICA was utilized to identify and remove artifacts caused by eye drift, muscle activity, and head movements. 6. Data averaging: All data trials were overlaid and averaged to obtain a representative signal.

The processing of the above-mentioned EEG data can be accomplished using the MATLAB toolbox called EEGLAB. Alternatively, one can implement data scripts in MATLAB for processing. The analysis of EEG data typically involves both time-domain and frequency-domain analyses, such as event-related potential analysis, amplitude and phase analysis, spectral energy analysis, and time-frequency analysis using wavelet transforms. The mentioned analyses can provide insights into the impact of high-altitude low-oxygen environments on neural signals in the brain.

### Behavioural results

**Fig. 4:**
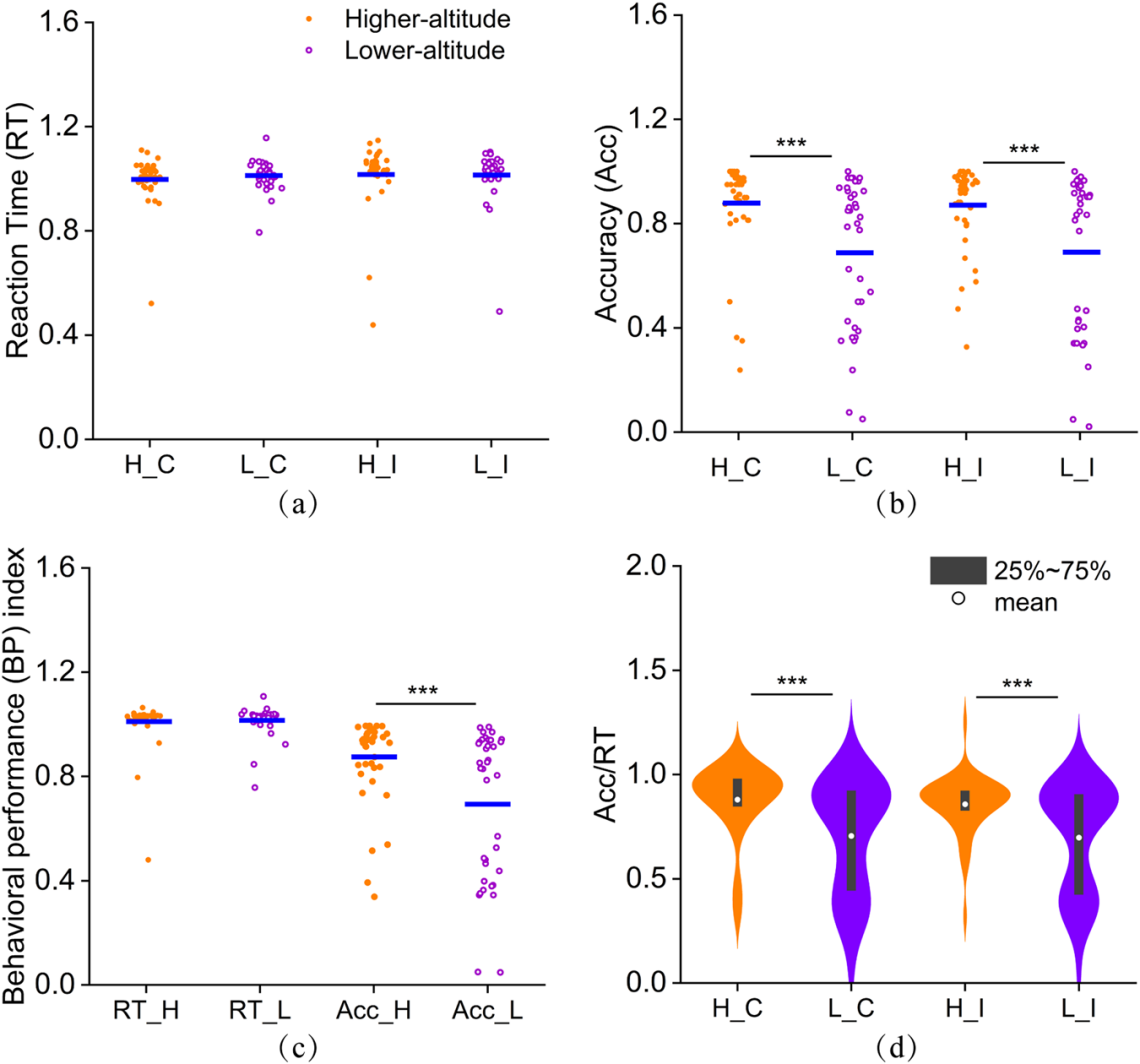
Analysis of behavioural data. Figure (**a**), (**b**) and (**c**) are the statistics of response time (RT) and accuracy (ACC) for all participants, and Figure (**d**) is the index of ACC/Time for the consistent stimuli (“C”) and conflicting stimuli (“I”) at high/low-altitudes.

The statistics of RT and ACC for participants while performing the Stroop tasks at high/low-altitudes (Fig. 4). Orange dots represent participants at high-altitudes, while purple circles represent participants at low-altitudes. Individuals who underwent hypoxic preconditioning at high-altitudes exhibited similar reaction times to those who underwent preconditioning at low-altitudes. However, their reaction ACC significantly improved, leading to superior behavioural performance when faced with conflicting stimuli^13,14^. The RTs for participants at both the high/low-altitudes are clustered at approximately 1 s, with no statistically significant difference between the two altitudes. The ACC of the high-altitude group was higher than that of the low-altitude group, with the difference between the two altitudes being extremely significant as determined by one-way ANOVA (p < 0.001). The ACC data of participants at low-altitude were more scattered, with approximately 39.5% of participants responding with an ACC of less than 0.6. In contrast, only approximately 10.5% of participants at the high-altitude had an ACC of less than 0.6. The ACC/RT index of the high-altitude group was significantly superior to that of the low-altitude group (p < 0.001), both in the “C” tasks and the “I” tasks. Further analysis revealed that for the “C” tasks, approximately 90% of participants at the high-altitude and 63% of participants at low-altitude had an ACC/RT ratio greater than 0.6. For the “I” tasks, these proportions were 93% and 62%, respectively.

### fNIRS results

**Fig. 5:**
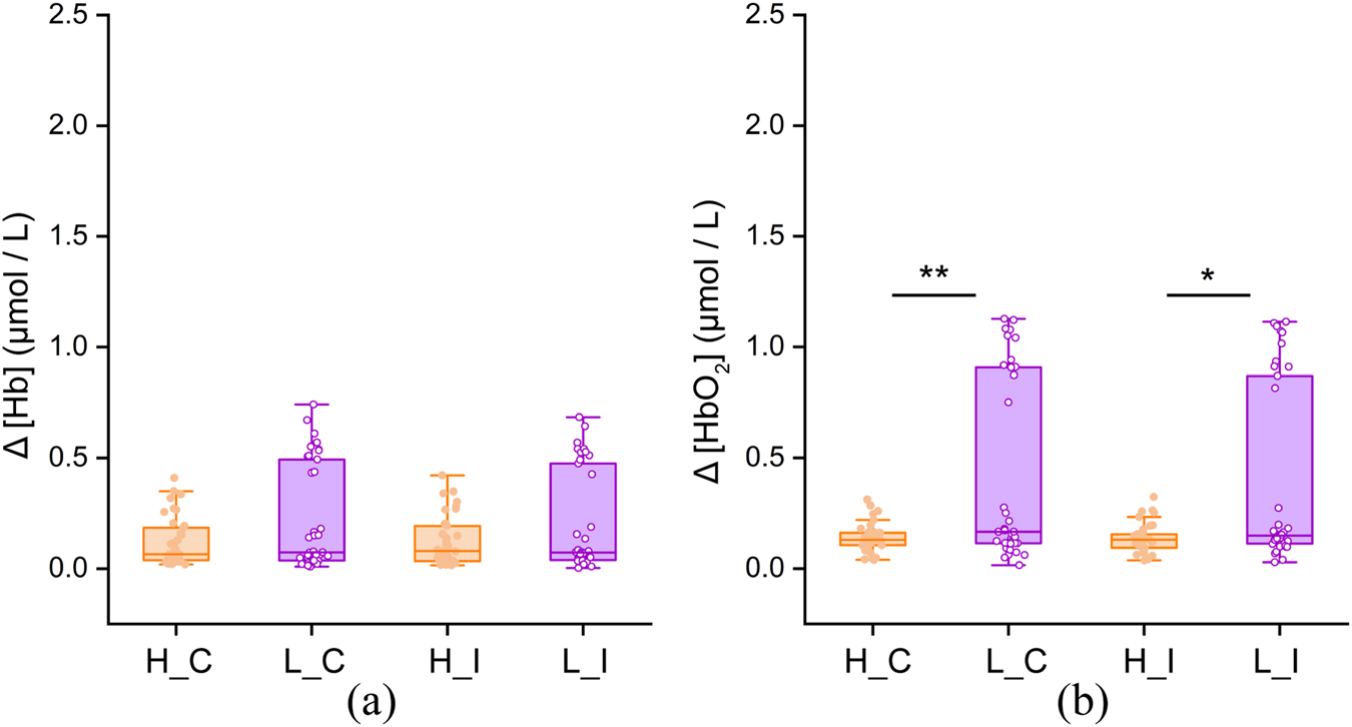
Statistics of prefrontal hemodynamics: (**a**) Δ[HbO2]; (**b**) Δ[Hb].

The Δ[HbO2] in the prefrontal lobe of the low-altitude group was significantly higher than that of the high-altitude group (one-way ANOVA, p < 0.05) (Fig. 5). Resulting from the elevation of altitude, the average Δ[HbO2] among all participants decreased by approximately 50%. Approximately 31.6% of participants at low-altitude had Δ[HbO2] < 0.5μmol/L, whereas only approximately 7.9% at the high-altitude had Δ[HbO2] < 0.5μmol/L. When comparing the between-group difference during the “C” and “I” tasks, the “C” task data showed a much higher level of significance (one-way ANOVA: “C”, p < 0.01; “I”, p < 0.05). Although the difference between the two altitudes in Δ[Hb] appeared to be nonsignificant, it was still evident that 28.9%-31.6% of low-altitude participants had Δ[HbO2] > 0.5μmol/L, while values for only 2.6% of high-altitude participants fell within this range.

### EEG results

**Fig. 6:**
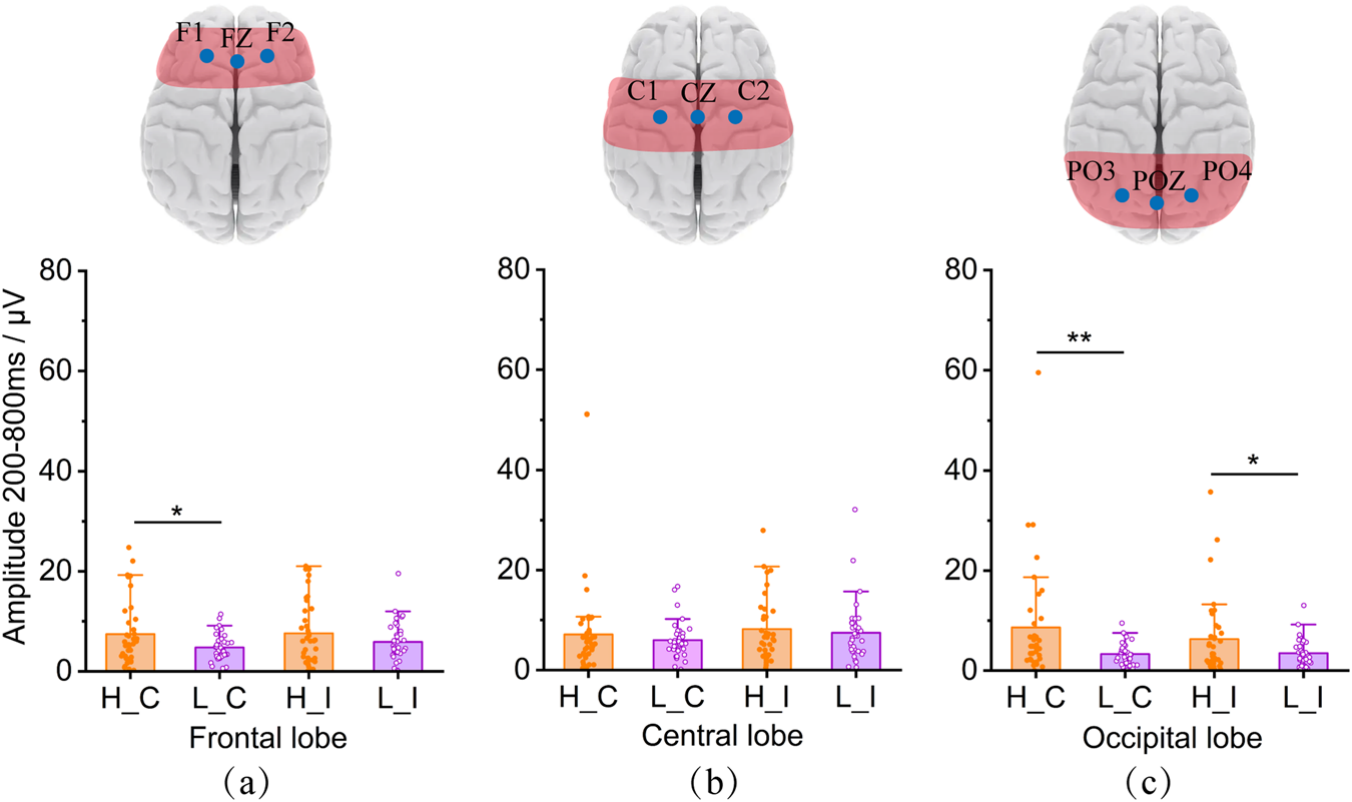
Comparison of EEG amplitudes. Figure (**a**) frontal lobe (F1, FZ, F2), (**b**) central lobe (C1, CZ, C2), and (**c**) occipital lobe (PO3, POZ, PO4).

We validated the availability of EEG data in both the time domain and frequency domain. Furthermore, we verified the quality of the provided data through specific analysis results. In high-altitude environments, the hemoglobin level in the human brain decreases, leading to alterations in attention and judgment when responding to stimuli. To further investigate, we analyzed the alterations in characteristic EEG signals. The comparisons of EEG amplitude values between the high/low-altitudes during the period of 200–800 ms (Fig. 6). The occipital lobe showed the most pronounced disparities between the two test altitudes. The high-altitude group exhibited higher amplitudes and a broader range of activation than the low-altitude group, particularly during the “C” tasks.

**Fig. 7:**
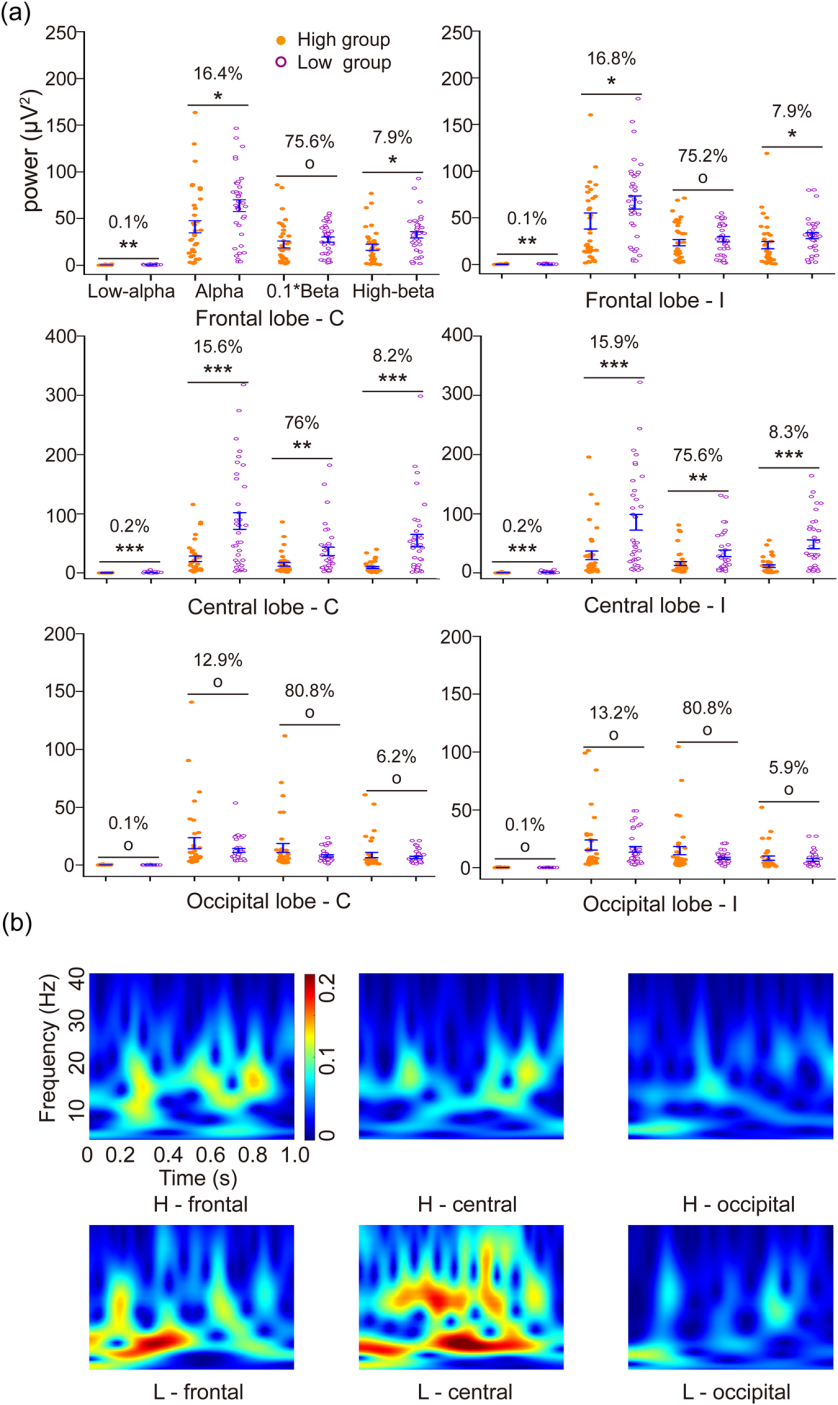
(**a**) The sub-band extraction of EEG spectrum power. (**b**) The colour map of EEG analysis in the time-frequency domain. The x-axis represents the time series (0-1 s), the y-axis represents the frequency (8–35 Hz), and the colour depth indicates the intensity of EEG activation.

The frequency spectrum densities are displayed across different EEG frequency bands (Fig. 7). The x-axis represents the frequency bands (Low-α, 0.1–8 Hz; Α, 8–14 Hz; Β, 14–30 Hz; High-β, 30–40 Hz), while the y-axis represents EEG power. The percentage indicates the ratio of EEG density within a specific frequency band to the total energy (0.1–45 Hz). To fit all the data within a suitable range on the y-axis, we plotted the data multiplied by 0.1*β. For the low-altitude group, the EEG activation in the α-band in the frontal and central lobes exhibited a bar-shaped distribution (Fig. 7b). The EEG activation was more dispersed in the time-frequency domain compared to the high-altitude. In contrast, the high-altitude group showed concentrated EEG activation in the β-band within the 200–800 ms interval. Both altitude groups exhibited stronger EEG activation in the frontal and central lobes, while the occipital lobe showed relatively weak activation.

## Data Availability

Behavioral data is recommended to be opened using MATLAB 2022b. According to the provided data instructions, you can directly process the data to obtain reaction times and accuracy for each participant. However, data from participants with extremely low accuracy should not be included in the effective statistical range. For fNIRS data, it is advisable to open it using MATLAB 2022b. Design a band-pass filter to filter the raw data before proceeding with further analysis. When processing EEG data, it is necessary to preprocess the data for each participant using the MATLAB plugin eeglab. Specific instructions can be found in the official eeglab user manual. We also provide reference data processing scripts, which can be obtained on GitHub. No custom code was used during this study.
